# Therapeutic evaluation of arterio-portal fistula-related gastroesophageal variceal bleeding

**DOI:** 10.18632/oncotarget.16579

**Published:** 2017-03-25

**Authors:** Xiaoquan Huang, Wen Zhang, Shiyao Chen, Chengfeng Liu, Ruofan Sheng, Feng Li, Jian Wang, Jianjun Luo, Pengju Xu

**Affiliations:** ^1^ Department of Gastroenterology, Zhongshan Hospital, Fudan University, Shanghai, China; ^2^ Department of Interventional Radiology, Zhongshan Hospital, Fudan University, Shanghai, China; ^3^ Department of Radiology, Zhongshan Hospital, Fudan University, Shanghai, China

**Keywords:** combination treatment, portal hypertension, intrahepatic arterio-portal fistula, upper gastrointestinal bleeding

## Abstract

**Background & Aims:**

Intrahepatic arterio-portal fistula is an uncommon etiology of portal hypertension, which presents diagnostic and therapeutic challenges. This study aimed to assess the efficacy and outcomes of gastroesophageal variceal bleeding caused by arterio-portal fistula using different therapeutic approaches.

**Methods:**

Medical records of 451 consecutive patients with arterio-portal fistula were reviewed from January 1, 2009, to July 15, 2016, and patients suffered variceal bleeding were eligible for the study.

**Results:**

Among 57 patients with arterio-portal fistula, hepatocellular carcinoma was existed in 61.4% patients. A combination of radiological intervention and endoscopic treatment was performed in 8 (14.0%) patients; the remainder were treated using radiological intervention alone (*n* = 20, 35.1%), endoscopic treatment alone (*n* = 18, 31.6%), or without any intervention (*n* = 11, 19.3%). No patient died in the combination group, while 20 patients in the single-treatment group and 6 in the untreated group died during follow-up. A significant difference in the survival rate was found between the combination group and the other two groups. Treatment selection between combination and untreated groups was the only parameter significantly associated with survival (*p* = 0.002).

**Conclusions:**

For patients diagnosed with arterio-portal fistula, combination treatment is the most optimal strategy in managing variceal bleeding, especially in patient with severe type of fistula.

## INTRODUCTION

Intrahepatic arterio-portal fistula (IAPF) is an uncommon etiology of portal hypertension, which presents diagnostic and therapeutic challenges. Because of the communication between the hepatic artery and the portal vein, intrasinusoidal portal hypertension may result in ascites, hepatic encephalopathy, and varices with life-threatening gastrointestinal hemorrhage [1][2]. Fewer than 10% cases of IAPF are congenital, and most cases of IAPF are secondary to liver tumors, interventional hepatic procedures, cirrhosis, and so on [3]. The diagnosis of IAPF is usually made accidentally on radiographic evaluation of the portal circulation. However, these patients are easy to be misdiagnosed or miss diagnosed at the first presentation of upper gastrointestinal bleeding (UGIB). Limited data are available on the management of gastroesophageal variceal bleeding in these patients. At present, hepatic arteriography is the gold standard in diagnosing IAPF and may also play a therapeutic role [4]. A review of patients with IAPF reported that transcatheter arterial embolization (TAE), an interventional radiological procedure, is the predominant therapeutic choice because of its low invasiveness [5]. Nevertheless, some of the patients suffered refractory variceal bleeding after radiologic intervention and even underwent surgical ligation or resection of the hepatic lobe [1][2]. Endoscopic treatments including *N*-butyl-2-cyanoacrylate injection for gastric varices and endoscopic ligation or sclerotherapy for esophageal varices have never been reported as the therapeutic approach to UGIB in patients with IAPF.

Therefore, the aim of this retrospective study was to evaluate the efficacy and outcomes of gastroesophageal variceal bleeding caused by IAPF using different therapeutic approaches.

## PATIENTS AND METHODS

### Study design

This was a retrospective, tertiary hospital–based study. Between January 1, 2009, and July 15, 2016, 451 consecutive patients were diagnosed as IAPF(s) by contrast-enhanced computed tomography (CT) at Zhongshan Hospital, Fudan University, Shanghai, China. Clinical data were analyzed from prospectively collected data on the Portal Hypertension database, and from medical records. In all cases, the diagnosis of gastroesophageal varices was made by either upper digestive endoscopy or CT scanning. All patients who suffered variceal bleeding before or after the diagnosis of IAPF were eligible for the study.

The study protocol was conducted conforming to the guidelines of the Declaration of Helsinki, as reflected in the approval granted by the Institutional Human Research Committee.

Patients with IAPF were categorized according to the therapies they received. The patients in the single-treatment group were given either endoscopic treatment or radiologic intervention, whereas those in the combination treatment group received both endoscopic treatment and radiologic intervention in any sequence. The patients in the untreated group did not receive endoscopic or radiologic interventions.

The study initiation was set on the day when patients experienced the first variceal bleeding episode, and the end point was July 31, 2016. All the participants were followed up via phone calls, outpatients’ clinic visits, and chart reviews. The primary outcome of the study was all-cause death, and the secondary outcome was the incidence of rebleeding after treatment among patients receiving interventions.

### Definition

**Figure 1:**
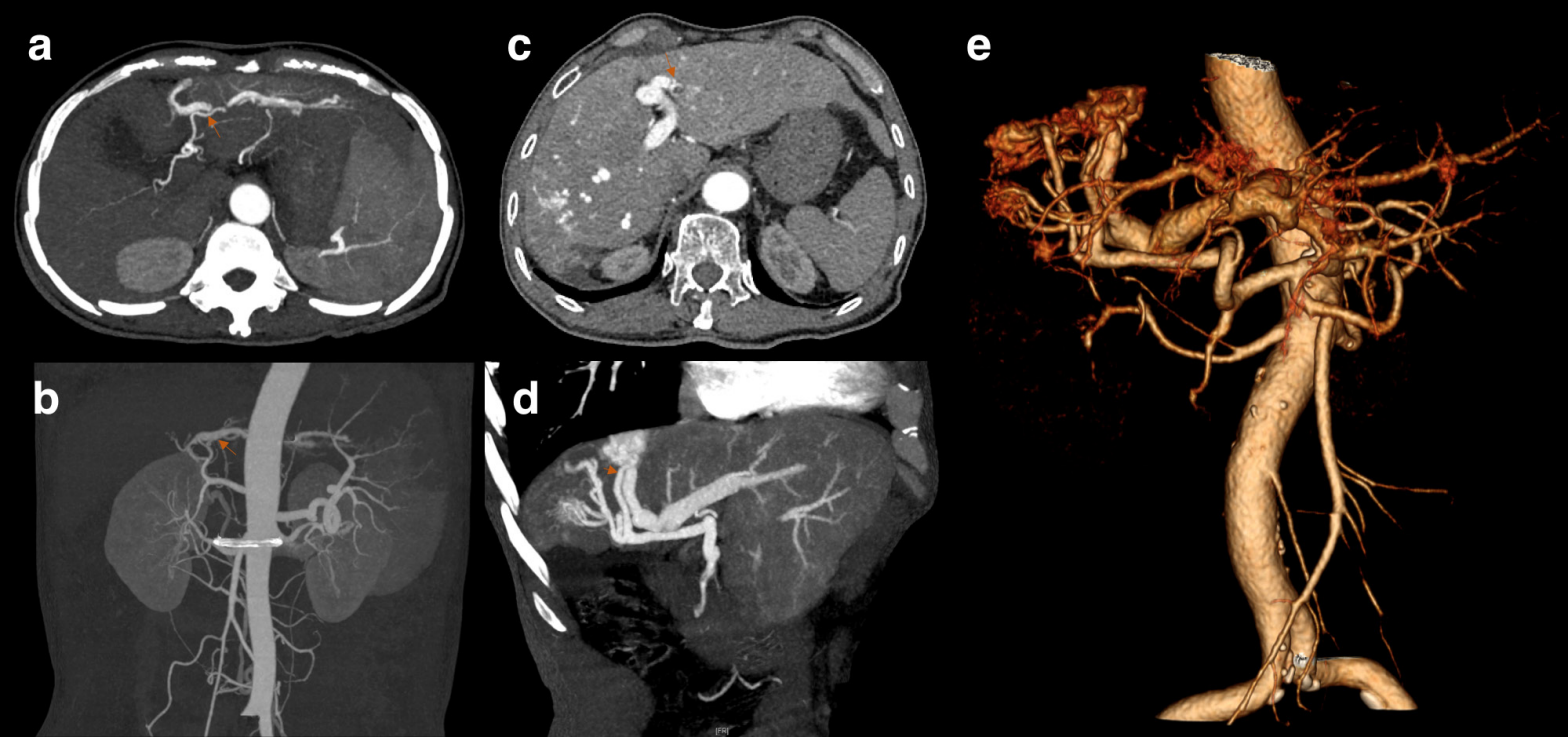
Figure a and b are from the same patient and demonstrate the mild type of IAPF **a**. The arterial phase of dynamic CT shows early enhancement of transient, peripheral, small hepatic vein branches. **b.** Maximal intensity projection (MIP) image of the portal trunk in the arterial phase shows the mild type of fistula. Figure c, d, and e are from the same patient and demonstrate the severe type of IAPF. **c.** Multiple fistulas developed in both central and peripheral areas. **d.** A large fistula was seen in the MIP image. **e.** Volume-rendered images show multiple fistulas in a severe type of IAPF. (Red arrows for concomitant hepatic arteries).

Rebleeding was defined as new onset of hematemesis, coffee-ground vomitus, hematochezia, or melena according to the guideline [6]. Early diagnosis of IAPF was described as the diagnosis of IAPF before or 7 days within the first bleeding episode. A peripheral type of IAPF was defined as a fistula located under the right/left branches of the portal vein, and a central type of IAPF was defined as a fistula located in the main portal vein. However, in a diffuse type of IAPF, fistulas involved both the main portal vein and branches in the liver. Based on the severity, IAPFs in the present study were classified into two groups, mild and severe, by the extent of the early enhancement of peripheral portal vein branches and the presence of wedge-shaped, transient peripheral areas of enhancement in the arterial phase, as previously described by Guzman and Choi et al [7][8]. Figure 1 demonstrates the CT angiography of patients with IAPF.

### Endoscopic treatment of varices

The patients received endoscopic treatment of variceal bleeding for either management of acute variceal bleeding or secondary prophylaxis of rebleeding. Endoscopic procedures were performed using Olympus-240/260 Gastroscopy (Olympus, Tokyo, Japan) under propofol sedation. As for esophageal varices, endoscopic ligation or sclerotherapy was performed by two experienced endoscopic specialists. Endoscopic ligation was applied using the Six-Shooter Multi-Band Ligator (Cook Endoscopy, Inc., NC, USA) at 1 cm above the Z-line in a spirally ascending fashion, with no more than six bands used per session. As for sclerotherapy, an intravariceal or paravariceal injection of lauromacrogol (10-30 mL per session; Tianyu Pharmaceutical Co., Ltd., Xi’an, China) was started above the Z-line and proceeded until all the visible esophageal varices were treated. Usually, endoscopic ligation is the primary selection and sclerotherapy is performed during follow-up endoscopy in those small esophageal varices which ligation is impossible. As for gastric varices, the “sandwich technique” was used for an N-butyl-2-cyanoacrylate (Compont Medical Adhesive, 0.5 ml/tube; Beijing Compont Medical Devices Co., Ltd., Beijing, China) injection. After flushing the injection needles (NM-200L-0423; Olympus, Tokyo, Japan) with an isotonic sodium chloride solution, the gastric varices were injected with lauromacrogol (4ml), cyanoacrylate (0.5-5ml), and lauromacrogol (4ml) again. The needle sheath was held at the puncture site for 3-4s to prevent leakage of cyanoacrylate. Follow-up endoscopy was performed at an interval of no less than 2 months and treatment was repeated until complete obliteration was achieved.

### Radiologic interventions of fistula

Transarterial embolization (TAE) was applied as a radiologic intervention for treating IAPF. Under local anesthesia, the transfemoral approach was routine. Selective angiography of the celiac axis and hepatic artery was performed with a 5F RH catheter (Cook, Bloomington, IN, USA). The embolic agent, microspheres (Embosphere Microspheres, BioSphere Medical, diameters ranging from 500 to 1200 μm) and irregular PVA particles (500–1000 μm, multiple vendors), multiple Tornado embolization microcoils (Cook, Bloomington, IN, USA), *N*-butyl-2-cyanoacrylate (Compont Medical Adhesive; Beijing Compont Medical Devices Co., Ltd., Beijing, China, 0.5ml), or gelatin sponge particles (100-1400μm) were selected by the velocity of the flow of fistula-feeding artery(s) and the size and location of the fistula. The end point was determined by the satisfactory occlusion and disappearance of the fistula on angiography. Multiple interventions may be necessary for large-size fistulas.

### Statistical analysis

Continuous variables were expressed as the mean ± standard error of mean or median (range) and compared using the Student’s *t* test. Categorical variables were described with constituent ratios and compared using the chi-square or Fisher’s exact test. The Kaplan-Meier method with log-rank test was used to estimate the cumulative probability of rebleeding and death. The Cox regression model was used to evaluate potential risk factors for survival. The bootstrap technique was performed, and the sample was set at 500. Model selection was guided by the results of univariate analyses (*P* < 0.10), and the best statistical fit was identified through the stepwise algorithm. Calculations were made using SPSS 23.0 software (SPSS, IL, USA). All statistical analyses were two-sided tests (*P* < 0.05).

## RESULTS

### Patient characteristics at first bleeding

**Figure 2:**
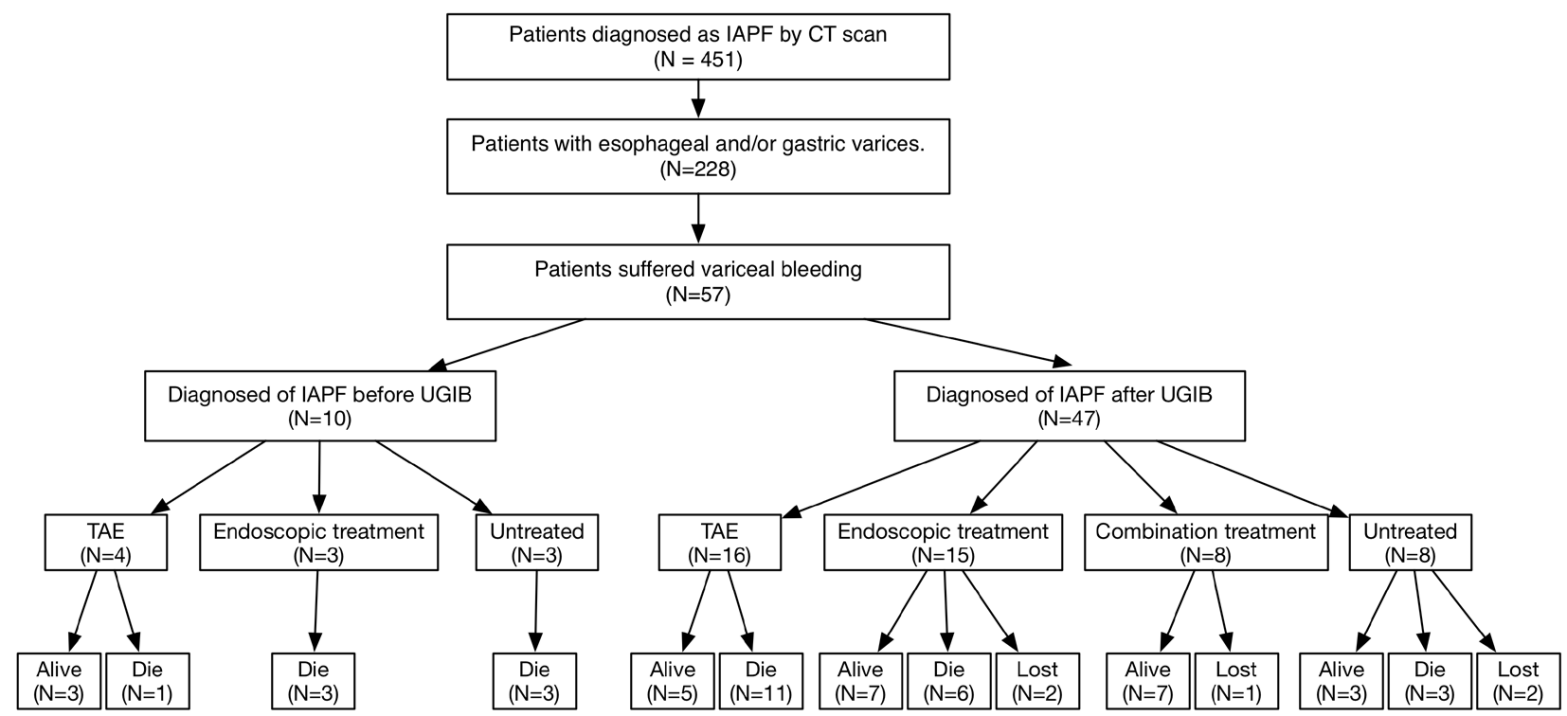
Study flowchart showing patients’ inclusion and outcomes

Among 451 consecutive patients diagnosed with IAPF by CT scanning, 228 (50.6%) had esophageal and/or gastric varices and 57 (12.6%) suffered from cirrhotic variceal bleeding episode. The mean age at first bleeding was 54.3±1.7 years (range, 22−79), and 70.2% of the patients were males. Hepatocellular carcinoma (HCC) was existed in 35 (61.4%) patients. The median period retrospect to the first episode of variceal bleeding until the end point was 281 days (range, 2−3361). Most of the patients had mild (*n* = 20, 35.1%) to severe (*n* = 26, 45.6%) ascites at presentation of variceal bleeding. The study flowchart and outcomes are shown in Figure 2. The characteristics of these patients with IAPF and the laboratory values are listed in Table 1.

**Table 1:** Patient demographics and baseline clinical and biochemical characteristics

Variables	Total (*n* = 57)
Age at first bleeding (year)	54.3 ± 1.7 (2279)
Age at diagnosis of IAPF	55.0 ± 1.7 (2380)
Early diagnosis of IAPF (yes/no)	10 (17.5%)/47 (82.5%)
Sex (male/female)	40 (70.2%)/17 (29.8%)
HBV (positive/negative)	40 (70.2%)/17 (29.8%)
Etiology of IAPF	
-HCC	35 (61.4%)
-Cirrhosis	18 (31.6%)
-AVM	4 (7.0%)
Hemoglobin (g/dL)	8.1 ± 0.3 (3.8−12.7)
White blood cell count (109/L)	4.4 ± 0.3 (0.86−16.3)
Thrombocytes (103/L)	94.5 ± 7.8 (29−275)
Bilirubin (µmol/L)	25.3 ± 3.9 (5.1−191.6)
Albumin (g/L)	30.7 ± 0.7 (19−43)
Prothrombin time (s)	14.5 ± 0.4 (11.3−25.2)
INR	1.2 ± 0.03 (0.98−2.17)
Creatinine (µmol/L)	93.3 ± 16.2 (30−826)
Child-Pugh Class (A/B/C)	10 (17.5%)/35 (61.4%)/12 (21.1%)
MELD Score	12.5 ± 0.1 (7−15)
Splenectomy	7 (12.3%)
Portal vein embolus	35 (61.4%)
AFP (abnormal/normal)	24 (42.1%)/33 (57.9%)
Ascites	
-Absent	11 (19.3%)
-Mild	20 (35.1 %)
-Severe	26 (45.6%)
Hepatic encephalopathy	
-Absent	48 (84.2%)
-Mild	8 (14.0%)
-Severe	1 (1.8%)
Severity of IAPFs (mild/severe)	25 (43.9%)/32 (56.1%)
Types of IAPFs	
-Peripheral type	26 (45.6%)
-Central type	28 (49.1%)
-Diffuse type	3 (5.3%)

Continuous variables are expressed as mean ± standard error of mean with range in parentheses.

The Child-Pugh score ranges from 5 to 15: class A (5−6 points), class B (7−9 points), and class C (10−15 points).

Abnormal AFP value is defined as >20.0 ng/mL.

AFP, Alpha fetal protein; AVM, arteriovenous malformation; HBV, hepatitis B virus; INR, international normalized ratio; MELD, Model for End-stage Liver Disease.

### Procedural characteristics

IAPFs were diagnosed by contrast-enhanced CT scanning. Ten patients were diagnosed with IAPF before UGIB, while the other 47 patients were diagnosed with IAPF after variceal bleeding. Seven patients received splenectomy at the first presentation of UGIB before the diagnosis of IAPF. The interval from the first episode of bleeding until the diagnosis of IAPF as a possible etiological factor for portal hypertension was 21 days (range, −538 to 5438). A combination of radiological interventions and endoscopic treatment was performed in 14.0% (*n* = 8) of patients, whereas the remainder were treated using radiological intervention alone (*n* = 20, 35.1%), endoscopic treatment alone (*n* = 18, 31.6%), or without any interventions (*n* = 11, 19.3%). All the 28 patients received radiological interventions were confirmed IAFP by hepatic arteriography.

Eighteen patients received solely endoscopic treatments with an average of 1.7 (range, 1−5) procedures, and 20 patients received only TAE approaches with the mean of 1.5 (range, 1−4) procedures. Four patients first received endoscopic procedures before the diagnosis of IAPF, and three of them switched to TAE at the moment of finding the existence of IAPF. The other one chose not to receive TAE treatment until the second severe variceal bleeding and had the hepatic venous pressure gradient measurement of 22 mmHg. Four patients had their fistula embolized first; three of them suffered recurrent variceal bleeding and thus received the endoscopic treatment, and the other one received combination endoscopic treatment for secondary prophylaxis of variceal bleeding.

### Primary and secondary end points

#### Overall survival

**Figure 3:**
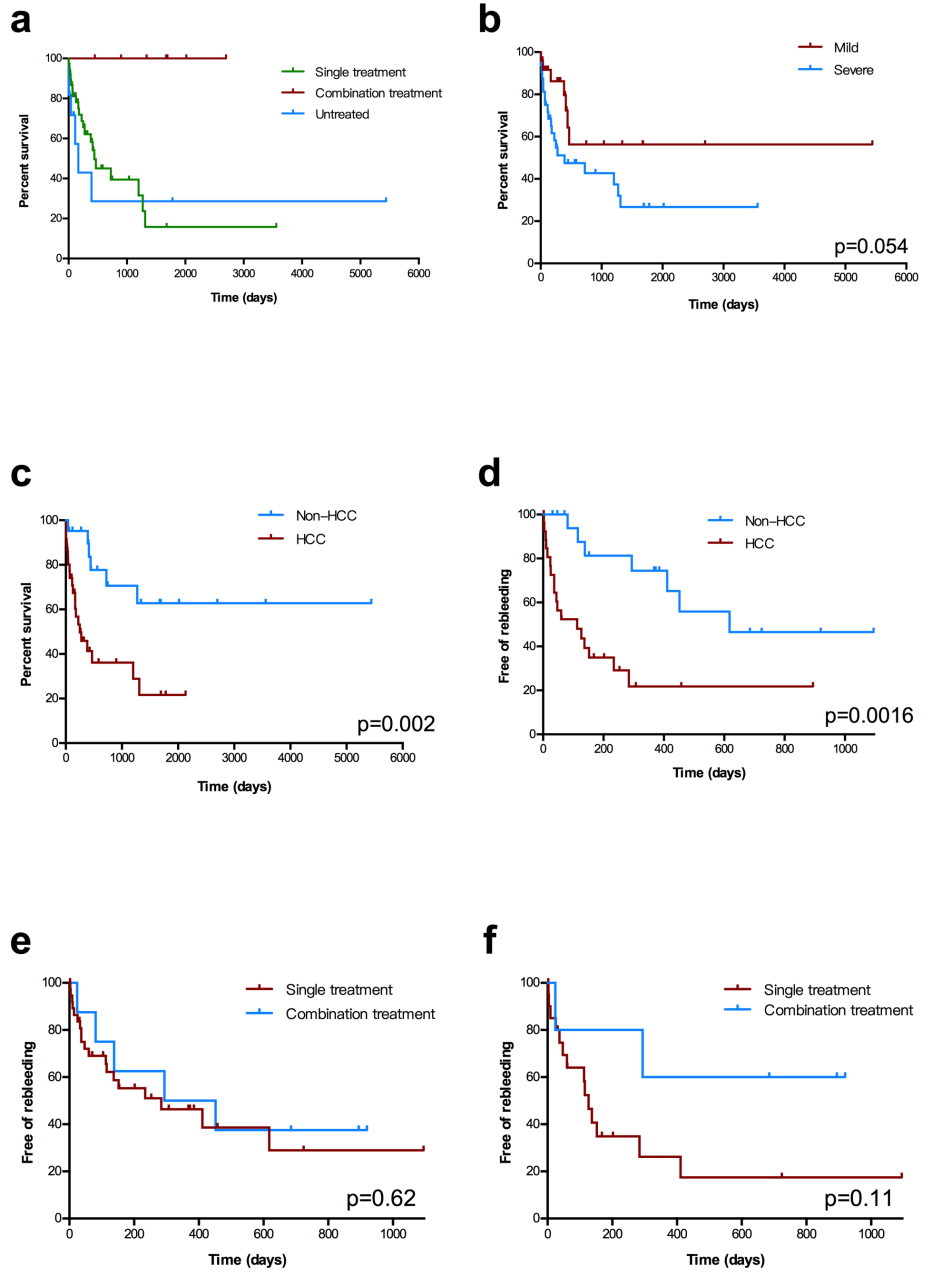
Kaplan−Meier estimates of overall survival and rebleeding **a.** Overall survival stratified by the three groups. **b.** Overall survival stratified by the severity of IAPFs. **c.** Overall survival stratified by patients with or without Hepatocellular Carcinoma (HCC). **d.** Three-year rebleeding rate stratified by with or without HCC in patients received treatment **e.** Three-year rebleeding rate in the two interventional groups in all patients. **f.** Three-year rebleeding rate among patients with severe IAPF in the two interventional groups.

A total of 21 (55.3%) patients in the single-treatment group [hypovolemic shock secondary to UGIB (*n* = 10); liver failure (*n* = 11)] and 6 (54.5%) patients [hypovolemic shock secondary to UGIB (*n* = 2), liver failure (*n* = 2), multi-organ dysfunction syndrome (*n* = 1), and unknown (*n* = 1)] in the untreated group died during the follow-up period. Four patients (three in the single-treatment group and one in the untreated group) underwent liver transplantation and were alive without further episodes of variceal bleeding. The cumulative survival rate at 1, 2, and 3 years was 100%, 100%, and 100% in the combination treatment group, 63%, 45%, and 39% in the single-treatment group, and 43%, 29%, and 29% in the untreated group. A significant difference in the survival rate was found between the combination treatment group and the other two groups (Figure 3a).

The results of Cox analysis are shown in Table 2. Based on 500 bootstrap samples, the univariate Cox regression analysis showed that concurrent HCC (*P* = 0.004), Child-Pugh Class (*P* = 0.002), severity of IAPFs (*P* = 0.040), portal vein embolus (*P* = 0.090), treatment selection (single treatment vs. untreated, *P* = 0.623; combination treatment vs. untreated, *P* = 0.004), and early diagnosis of IAPFs (*P* = 0.014) showed a trend toward survival. In the multivariate analysis, treatment selection between the combination treatment and untreated groups was the only parameter significantly associated with survival (*P* = 0.002).

**Table 2:** Univariate and multivariate analyses of overall survival

Variable	OS			
	Univariate analysis		Multivariate analysis	
	95% confidence interval	*P* value	95% confidence interval	*P* value
Age at first bleeding (<55 y vs. ≥55 y)	(−1.110) 0.441	0.425		
Sex (male vs. female)	(−1.837) 0.164	0.152		
HCC	0.641−2.701	0.004	(−0.020)−3.192	0.112
HBV	0.109−1.985	0.026		
Child-Pugh class (A vs. B vs. C)	0.462−1.695	0.002	(−0.257)−1.827	0.126
Severity of IAPFs	(−0.047)−1.876	0.040	(−0.459)−2.288	0.257
Portal vein embolus	(−0.126) 1.638	0.090	(−0.737) 2.086	0.393
Treatment selection				
Single vs. untreated	(−1.875−0.967	0.623	(−2.040)−1.203	0.661
Combination vs. untreated	(−15.947)−(−12.861)	0.004	(−16.224) (−11.658)	0.002
Diagnosis of IAPFs before or 7 days within bleeding	(−2.370)−0.172	0.014	(−3.043)−0.502	0.236

For the endpoint overall survival, patients with IAPF were divided into two groups based on the severity of their fistula. The cumulative survival rate at 1, 2, and 3 years was 86%, 56%, and 56% in the mild-IAPF group (*n* = 25) and 50%, 47%, and 42% in the severe-IAPF group (*n* = 32) (*p* = 0.054, Figure 3b). For IAPF-patients with or without HCC, the overall survival showed significance (*p* = 0.002, Figure 3c).

#### Rebleeding

The present study aimed at patients’ occurrence of rebleeding after receiving interventions. The free-of-rebleeding period was defined as the time interval between the date of first treatment and the first episode of rebleeding after treatment or follow-up to 3 years. Patients concomitant HCC had the higher risk of rebleeding after the interventions (*p* = 0.0016, Figure 3d). Nevertheless, the cumulative probability of remaining free of rebleeding in 3 years revealed no significant difference between the two interventional groups (*P* = 0.62, Figure 3e). The non-rebleeding rate at 6 months, and 1 and 2 years was 71%, 56%, and 43% in the combination treatment group and 50%, 41%, and 26% in the single treatment group, respectively.

Stratification analysis was performed on patients in the severe-IAPF group. The trend that combination treatment (*n* = 5) was superior to single treatment (*n* = 20) is shown in Figure 3f.

Further analysis was conducted on the patients receiving interventions, who were divided into two groups according to the first procedure they underwent. Twenty-two of them first received endoscopic treatment, and the other 24 patients first underwent TAE. A total of 26 patients developed rebleeding. Twelve (54.5%) in the group first received endoscopic treatment, and 14 (56.0%) first received TAE procedures. Time to rebleeding and outcomes are listed in Table 3.

**Table 3:** Rebleeding and outcomes in the group that first received endoscopic treatment and the group that first underwent TAE stratified by the severity of IAPF

	Endoscopic treatment first (*n* = 22)		TAE first (*n* = 24)	
Severity of IAPF	Mild	Severe	Mild	Severe
Rebleeding	5 (22.7%)	7 (31.8%)	3 (12.5%)	11 (45.8%)
7-day rebleeding	0	2 (9.1%)	0	0
3-month rebleeding	2 (9.1%)	1 (4.5%)	3 (12.5%)	5 (20.8%)
6-month rebleeding	1 (4.5%)	2 (9.1%)	0	3 (12.5%)
12-month rebleeding	1 (4.5%)	0	0	2 (8.3%)
Interval between first treatment and rebleeding (days)*	138 (10, 618)	137 (2, 1270)	253 (10, 1209)	115 (9, 920)
Death	1 (4.5%)	8 (36.4%)	4 (16.7%)	8 (33.3%)
7-day death	0	1 (4.5%)	0	0
3-month death	0	3 (13.6%)	1 (4.2%)	2 (8.3%)
1-year death	0	1 (4.5%)	0	5 (20.8%)
3-year death	1 (4.5%)	1 (4.5%)	3 (12.5%)	0
Survival time (days)*	426 (30, 2699)	264 (2, 1272)	402 (37, 1680)	277 (41, 3560)

* Median, range.

## DISCUSSION

In this study conducted in a tertiary hospital famous for liver disease, the CT reports from more than 10,000 patients with liver disease and 451 patients diagnosed with IAPFs were screened. About half of the patients with IAPF had gastroesophageal varices with a 12.9% bleeding rate. The results showed that the combination of endoscopic treatment and TAE is the most optimal therapy.

Since the first IAPF case reported in 1892 [9], etiologies include trauma, iatrogenic complications of diagnostic and therapeutic procedures[10], congenital lesions, arteriovenous shunting within HCC, spontaneous development of a fistula, and so on [1]. In the present study, the major etiology of IAPFs is HCC, that existed in 61.4% of patients. In line with previous studies on IAPFs, it was found that ascites were the common manifestations of gastrointestinal bleeding while hepatic encephalopathy was less common [1]. Also, 46 patients in the present study had ascites while 26 patients presented with massive ascites.

The importance of secondary prophylaxis after variceal bleeding in patients with IAPF is considerably acknowledged, yet the therapeutic approaches are limited. Currently, no therapeutic strategy exists regarding the management of varices in patients with IAPF via endoscopy, and the predominant treatment in these patients is TAE. However, patients suffered refractory rebleeding after TAE procedures are common. Previous studies showed that long-standing IAPFs might be associated with structural liver changes, including vascular changes, minimal peripheral fibrosis, and conserved lobular architecture [11]. Radiologic interventions can only deal with the fistula-feeding vessel(s), but the sustained presence of gastroesophageal varices remains a high risk of variceal hemorrhage. Especially in patients with mild severity of IAPF, TAE can only reduce a small amount of portal pressure; the remaining varices have the high risk of rebleeding.

Combination treatment has the best efficacy especially in patients with severe IAPF, while single treatment using either endoscopic treatment or TAE is only slightly better than no treatment. The reasons may be that TAE simply embolizes the fistula and partially reduces the portal pressure added by the shunt. The original cirrhotic portal hypertension is still present and coexists with the gastroesophageal varices. Thus, the risk of rebleeding and death is not reduced. As for endoscopic treatment, however, it has the unsatisfactory outcomes in patients with HVPG larger than 20mmHg [12]. For these patients with IAPF, the existing arterial-venous shunt increases the portal pressure compared with the general cirrhotic patients. Hence, the efficacy of endoscopic treatment for variceal bleeding is not achieved. The symptoms of IAPFs are dependent on the severity of fistula, and the amount of blood shunted that increases portal pressure. Single endoscopic or TAE procedures have equivalent efficacy in IAPFs, which corresponds to their own efficacy. The combination of endoscopic and TAE treatment achieves the optimal efficacy by decreasing portal pressure through embolization of the fistula and alleviating the rebleeding risk by endoscopic treatment.

Remarkably, seven patients received splenectomy before the diagnosis of IAPF and 47 patients (82.5%) diagnosed with IAPF after the first variceal bleeding. It indicated that IAPFs were easy to be misdiagnosed in clinical practice. In Cox regression analysis, treatment selection between untreated and combination treatment groups is the only parameter that influences survival. It is essential to achieve a better prognosis by prompt CT angiography when variceal bleeding occurs and have the optimal treatment selection. The early detection of the existence of IAPF helps in thoughtfully considering these patients and providing them essential treatment.

The free-of-rebleeding period was similar in both the combination and single-treatment groups. That is, either TAE or endoscopic treatment could not prevent variceal bleeding. Further stratification analysis showed that no matter what the first receiving procedure was, the rebleeding rate and overall survival had no difference.

Endoscopic procedures, including esophageal ligation, sclerotherapy, and tissue adhesive injection, were widely recommended in managing gastroesophageal hemorrhage [6][13]. In patients with mild-severity IAPF, the outcome of endoscopic treatment was slightly better than that of TAE procedures because these patients were similar to other patients with cirrhotic variceal bleeding. However, no recommendation for the management of variceal hemorrhage in patients with IAPF was found. Liver transplantation is considered the final method of rescuing patients with unresectable collateralization [14]. Four patients in the present study underwent liver transplantation for their primary liver disease, and they were alive without further complications during follow-up. In our experience, most of the IAPFs could be well managed by radiological intervention and endoscopic procedures.

The present study had some limitations. First, in this retrospective study, different sample size among three groups and differences in the severity of IAPFs and disease might have influenced the results when comparing groups. With the bootstrap sampling Cox regression model, combination treatment appeared to be the best therapeutic strategy. Second, the study included patients with IAPF regardless of any clinical status; consequently, some of the patients in poor condition introduced bias into the overall survival.

Although IAPF is uncommon, it should be considered as one of the differential diagnosis when refractory variceal bleeding occurs. For those diagnosed with IAPF, a combination of endoscopic management and radiologic interventional procedures is the most optimal treatment to avoid secondary massive UGIB and improve prognosis, especially in patient with severe IAPF.

## Abbreviations

IAPF: Intrahepatic arterio-portal fistula
UGIB: Upper gastrointestinal bleeding
TAE: Transcatheter arterial embolization
CT: Computed tomography
HCC: Hepatocellular carcinoma
